# Proteome-Wide Effect of 17-β-Estradiol and Lipoxin A_4_ in an Endometriotic Epithelial Cell Line

**DOI:** 10.3389/fendo.2015.00192

**Published:** 2016-01-06

**Authors:** Jonathan A. Sobel, Patrice Waridel, Ilaria Gori, Manfredo Quadroni, Geraldine O. Canny

**Affiliations:** ^1^The Institute of Bioengineering, School of Life Sciences, Ecole Polytechnique Fédérale de Lausanne, Lausanne, Switzerland; ^2^Swiss Institute of Bioinformatics, School of Life Sciences, Ecole Polytechnique Fédérale de Lausanne, Lausanne, Switzerland; ^3^Protein Analysis Facility, University of Lausanne, Lausanne, Switzerland; ^4^The Francis Crick Institute, London, UK; ^5^Ecole Polytechnique Fédérale de Lausanne, Lausanne, Switzerland

**Keywords:** endometriosis, endometriotic epithelial cells, mass spectrometry, lipoxin A_4_, 17-β-estradiol, proteomic analysis, protein–protein interaction network, gene ontologies

## Abstract

Endometriosis affects approximately 10% of women of reproductive age. This chronic, gynecological inflammatory disease results in a decreased quality of life for patients, with the main symptoms including chronic pelvic pain and infertility. The steroid hormone 17-β Estradiol (E2) plays a key role in the pathology. Our previous studies showed that the anti-inflammatory lipid Lipoxin A_4_ (LXA_4_) acts as an estrogen receptor-alpha agonist in endometrial epithelial cells, inhibiting certain E2-mediated effects. LXA_4_ also prevents the progression of endometriosis in a mouse model via anti-proliferative mechanisms and by impacting mediators downstream of ER signaling. The aim of the present study was therefore to examine global proteomic changes evoked by E2 and LXA_4_ in endometriotic epithelial cells. E2 impacted a greater number of proteins in endometriotic epithelial cells than LXA_4_. Interestingly, the combination of E2 and LXA_4_ resulted in a reduced number of regulated proteins, with LXA_4_ mediating a suppressive effect on E2-mediated signaling. These proteins are involved in diverse pathways of relevance to endometriosis pathology and metabolism, including mRNA translation, growth, proliferation, proteolysis, and immune responses. In summary, this study sheds light on novel pathways involved in endometriosis pathology and further understanding of signaling pathways activated by estrogenic molecules in endometriotic epithelial cells.

## Introduction

Endometriosis affects approximately 176 million women worldwide (1). This estrogen-dependant, inflammatory disease is characterized by the presence of endometrial-like tissue outside the uterine cavity, mostly on the pelvic peritoneum and ovaries. Symptoms include severe dysmenorrhea, dyspareunia, dysuria, chronic pelvic pain, and infertility, resulting in a decreased quality of life and significant socioeconomic consequences for patients (2–4).

17-β Estradiol (E2), the most potent estrogen, plays a pivotal role in endometriosis development and progression and is produced in high quantities in endometriotic lesions (5). Lipoxin A_4_ (LXA_4_) is an anti-inflammatory and pro-resolving lipid mediator with several anti-inflammatory actions *in vitro* and *in vivo* (6, 7). We previously demonstrated that LXA_4_, which possesses a high degree of structural similarity with the weak estrogen estriol, is an estrogen receptor alpha agonist in endometrial epithelial cells (8). In these studies, LXA_4_ altered estrogen-regulated gene expression as well as functional parameters, notably proliferation, in human endometrial epithelial cells while also demonstrating antiestrogenic potential in a manner similar to that previously shown for estriol (9, 10). LXA_4_ also prevented the progression of endometriosis in a mouse model via anti-inflammatory and anti-proliferative mechanisms and by impacting mediators downstream of ER signaling, including epithelial-expressed molecules such as growth regulation by estrogen in breast cancer (11). As such, the proteomic changes induced by this eicosanoid in *in vitro* models of endometriosis warrants further study.

As the small size of peritoneal endometriotic lesions precludes the culture of sufficient numbers of endometriotic epithelial cells, we used the well-characterized, ER-positive 12Z endometriotic epithelial cell line, originally isolated from peritoneal lesions (12). These cells express inflammatory molecules and exhibit a migrating and invading potential and are therefore an ideal model to study molecular and cellular aspects of endometriosis (13–15).

In recent years, Mass spectrometry (MS) has become a powerful technology to carry out large-scale analyses of cellular proteomes, as it allows qualitative and quantitative analysis of complex mixtures. One widespread quantitative method is based on Isobaric Tags for Relative and Absolute Quantification (iTRAQ) (16), which allows the comparison of multiple combined samples by chemically labeling peptides after digestion of protein extracts. With this labeling, small amine-reactive isobaric mass tags are attached to the N-terminus and lysine residues of peptides. Quantification is then based on a reporter ion generated during peptide fragmentation within the mass spectrometer and specific to each treatment in the mixed sample. The intensities of the reporter ion fragments enable the measurement of relative peptide abundance, and thus of their corresponding proteins (17).

The combination of state-of-the-art experimental strategies and advances in computational methods enables the global study of cellular proteomes. Computer-aided data mining such as annotation enrichment analysis enables more efficient mapping of complex proteomics data to biological processes (18). Searching for precise Gene ontology (GO) terms allows comprehensive summary analysis of large data sets, and the recently developed Cytoscape software is an useful tool to visualize protein–protein or protein–annotation networks (19–21).

The goal of the present study was to perform proteomic profiling of endometriotic epithelial cells to compare and contrast responses to E2, LXA_4_, and both in combination. LC-MS/MS analyses were carried out for relative quantification of proteins between treatments based on an iTRAQ approach. Interactome data, GO terms and pathway annotations were used to create protein–protein and protein–annotation networks to generate new information on estrogen and lipoxin signaling.

## Materials and Methods

### Cell Culture

12Z endometriotic epithelial cells (from Dr. Michael Beste, Massachusetts Institute of Technology, Cambridge, MA, USA) were maintained at 37°C in humidified air containing 5% CO_2_ in phenol red-free DMEM F-12 (Sigma) supplemented with 10% charcoal stripped-fetal bovine serum (CSFBS) (Invitrogen), 1% penicillin/streptomycin, and 1% glutamine (Sigma, Switzerland).

### Sample Preparation

6 × 10^5^ cells were seeded in 100-mm dish and treated with either vehicle (denoted control), 10 nM E2, 100 nM LXA_4_, or 10 nM E2 and 100 nM LXA_4_ in combination for 24 h. All treatments were carried out in duplicate. After the incubation time had elapsed, cells were rinsed with PBS and harvested by centrifugation. The pellet was washed twice with PBS and resuspended in 100 μl homogenization buffer (8M Urea containing Protease inhibitors). Samples were then sonicated three times for 15 s to solubilize the proteins. Samples were subsequently centrifuged for 15 min at 13,000 rpm and the supernatants were transferred into clean Eppendorf tubes and stored at −20°C. Proteins were quantified using a Bradford protein quantification kit (BioRad, Switzerland). Protein lysates were reduced with 5 mM DDT (Applichem, Switzerland) for 30 min at room temperature and then alkylated with 20 mM iodoacetamide (Sigma, Switzerland) for 30 min at room temperature protected from light. Proteins were precipitated with ethanol-acetate and resuspended in TEAB buffer (500 mM tetraethyl ammonium bicarbonate pH 8). For each sample, 220 μg of proteins was digested overnight at 37°C with 5 μg of trypsin. Thereafter, samples were aliquotted and stored at −80°C.

The samples were labeled with iTRAQ™ reagents (Applied Biosystems) as follows: 12Z non-treated sample 1, iTRAQ reagent 113; 12Z E2-treated sample 1, iTRAQ reagents 114; 12Z LXA_4_-treated sample 1, iTRAQ reagents 115; 12Z E2 + LXA_4_-treated sample 1, iTRAQ reagent 116; 12Z non-treated sample 2, iTRAQ reagent 117; 12Z E2-treated sample 2, iTRAQ reagents 118; 12Z LXA_4_-treated sample, iTRAQ reagents 119; 12Z E2 + LXA_4_-treated sample 1, iTRAQ reagent 121.

After sample clean-up with Proxeon SCX StageTips, labeling was controlled by LC-MS/MS separately for each sample before mixing of the eight digests. The mixed samples were redissolved in 4M Urea with 0.1% Ampholytes pH 3–10 (GE Healthcare, Switzerland) and fractionated by off-gel focusing as previously described (22). The 24 fractions obtained were desalted on a microC18 96-well plate (Waters Corp., Milford, MA, USA), dried, and resuspended in 0.1% (*v*/*v*) formic acid, 3% (*v*/*v*) acetonitrile for LC-MS/MS analyses.

### LC-MS/MS Analyses

Extracted peptides were analyzed on a hybrid LTQ Orbitrap Velos mass spectrometer (Thermo Fisher Scientific, Bremen, Germany) interfaced to an Ultimate 3000 RSLC nano HPLC system (Dionex, Switzerland). Peptides were separated for 120 min on a reversed-phase nanocolumn Acclaim PepMap RSLC 100A (75 µm ID × 25 cm, 2 µm, Dionex) at a flow rate of 300 nl/min using a H_2_O: acetonitrile gradient method. Lock mass option was used for full MS scan recalibration with a polydimethylcyclosiloxane ion from ambient air (*m/z* 445.12003). In data-dependent acquisition controlled by Xcalibur 2.1 software (Thermo Fisher Scientific, Switzerland), the 15 most intense precursor ions detected in the full MS survey performed in the Orbitrap (range 300–1700 *m/z*, resolution 30,000 at *m/z* 400) were selected and fragmented. MS/MS was triggered by a minimum signal threshold of 3,000 counts, carried out with stepped relative collision energy between 40 and 50% with an isolation width of 2.1 amu. Only precursors with a charge higher than 1 were selected for HCD fragmentation, and fragment ions were analyzed in the Orbitrap at a resolution of 7,500. The *m/z* of fragmented precursors was then dynamically excluded, with a tolerance of 10 ppm, for 25 s. To identify peptides, MS/MS files were analyzed with Proteome Discoverer 1.3 (Thermo Fisher Scientific, Switzerland) using Mascot 2.3 (Matrix Science, UK) for database searching. Mascot was set up to search the SwissProt database^1^ restricted to human taxonomy (database release used was 2011_03, 20,234 sequences after taxonomy filter), using the decoy database search option. Trypsin (cleavage at K and R, not before P) was used as the enzyme definition. Mascot was searched with a fragment ion mass tolerance of 0.02 Da, a parent ion tolerance of 10 ppm, allowing one missed cleavage. Iodoacetamide derivative of cysteine and iTRAQ (eight-plex) modification of lysine and peptide N-terminal were specified as fixed modifications. Deamidation of asparagine and glutamine, oxidation of methionine, and acetylation of protein N-terminal were specified as variable modifications. A False discovery rate (FDR) filter of 1% was applied.

### Statistical Analysis

Quantitative protein values were calculated as the median of peptide reporter ion intensities. Proteins with at least one identified peptide with a high confidence (FDR <0.01) in both replicates were considered for quantitative analyses. The protein quantification was normalized using the Lowess function (23). The log_2_ ratio of the treatment value divided by the control value was calculated for each protein and for each replicate.

The local pooled error test (LPE) was computed for each comparison (E2 versus control, LXA_4_ versus Control, and LXA_4_ + E2 versus control) as previously described (24). The FDR-adjusted *p*-value was computed using the Benjamini–Hochsberg (BH) correction. In our analysis, we defined two sets of proteins of interest, one with LPE *p*-values <0.05 and a subset group with FDR-adjusted *p*-values <0.1. Calculations were performed using R statistical software version 2.10.0.

### Gene Ontology Analysis

Gene Ontology analysis was performed on significantly differentially expressed proteins for one or more treatments versus control. GO terms were analyzed using DAVID (18)^2^. Biological process annotations with a minimum set of two annotated proteins in our query and an enrichment *p*-value <0.05 were used. The results of this analysis were subsequently visualized in Cytoscape v3.2.0^3^ to model the protein–annotation network.

### Pathway Annotation Retrieval

KEGG Pathway annotations were retrieved using DAVID (18) with a minimum of two annotated proteins, which were significantly differentially expressed (LPE *p*-value <0.05) in E2 or LXA_4_/E2-treated cells. The results of this analysis were subsequently utilized in Cytoscape to model the protein–annotation network.

### Protein Network Analysis

Cytoscape software for network visualization was employed to build a protein–protein interaction network (20, 21) using physical interactions, pathway interactions, and genetic regulation interactions from all available sources within GeneMANIA (25). A maximum of 20 proteins were automatically added to our query by GeneMANIA in order to fill potential gaps in our network, using the guilt by association principle.

### Western Blotting

Cells were treated with indicated concentrations of E2, LXA_4_, and both in combination for 24 h in E2-free medium. After the incubation time had elapsed, cells were rinsed with PBS and harvested in 2× SDS sample buffer [125 mM Tris-HCl (pH 6.8), 4% SDS, 20% glycerol, 100 mM DTT, 0.01% bromophenol blue and protease inhibitors (Sigma-Aldrich, Switzerland)]. Thirty micrograms of total protein were loaded and separated on 10% sodium dodecyl sulfate (SDS)-polyacrylamide gels. Proteins were electrophoretically transferred onto nitrocellulose membrane (Biorad Laboratories, Switzerland). The membrane was blocked overnight at 4°C in a 5% fat-free dry milk solution in TBS-containing 0.05% Tween 20 (TBS-T) and subsequently incubated for 1 h at RT with a rabbit anti CSN5 antibody (Abcam, UK) diluted 1:2,000 in 5% fat-free dry milk solution in TBS-T or with a mouse anti β-actin antibody (Sigma Aldrich, Switzerland) diluted 1:8,000. After washing, the membrane was incubated for 1 h with HRP-conjugated anti-rabbit secondary antibody or anti-mouse secondary antibody at a 1:3000 dilution in 5% non-fat milk in TBS-T. Immunoreactive bands were visualized using chemiluminescence (PerkinElmer, Wellesley, MA, USA) and densitometric analysis was performed using ImageJ software.

## Results

**Figure 1:**
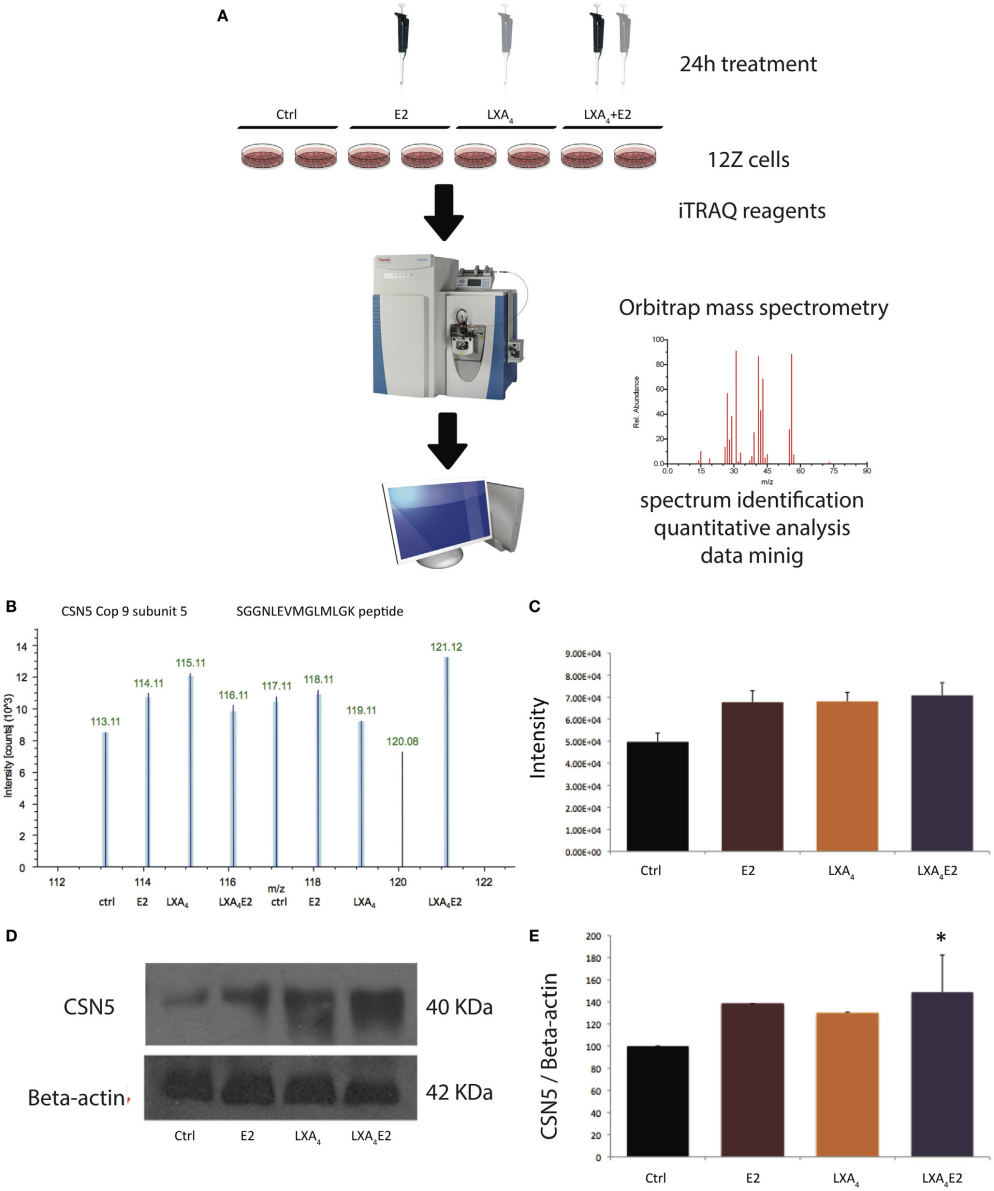
**Validation of MS data for COP9 subunit-5 (CSN5) by Western blotting**. **(A)** Schematic of the experimental procedure. **(B)** Representative reporter ion spectrum for CSN5 in 12Z cells, **(C)** mean intensity of reporter ions, **(D)** Western blot, and **(E)** resultant densitometric values. The bar graph of densitometric analysis shows the ratio of CSN5 to β-Actin protein expressed as a percentage. A representative blot from three independent experiments is shown. *p < 0.05 compared to vehicle-treated cells (ctrl). The reporter ion spectrum is derived from the detection of the SGGNLEVMGLMLGK peptide.

Our LC-MS/MS analyses (Figure 1A) resulted in the identification of 26,979 peptides (Table S1 in Supplementary Material). Proteins with at least one identified peptide in both replicates were considered for further quantification and analysis, culminating in 3,232 quantified proteins in Figure 1A. A representative spectrum of an iTRAQ quantitative analysis of CSN5 is represented in Figure 1B. CSN5 is a subunit of the COP9 signalosome implicated in diverse biological processes, such as signal transduction, development, and the cell cycle (26). CSN5 exhibited a log fold change of 0.3 between treatments versus control. To benchmark our quantitative approach, we carried out Western blotting for CSN5 (Figure 1D). The Western blot and MS data show a similar pattern (Figures 1C,E) and are highly correlated with a *R*^2^ linear regression value of 0.92. Densitometric analysis revealed that CSN5 levels were significantly increased in LXA_4_E2 conditions versus control (*p* < 0.05).

The variability of both replicates in each condition was checked using the logarithm of geometric mean intensity compared to the logarithm of the intensity ratio of the two replicates (Figure S1 in Supplementary Material). Approximately 95% of the protein log_2_ ratio values were between −0.5 and 0.5 in each condition, indicating a low variability between the two replicates.

**Figure 2:**
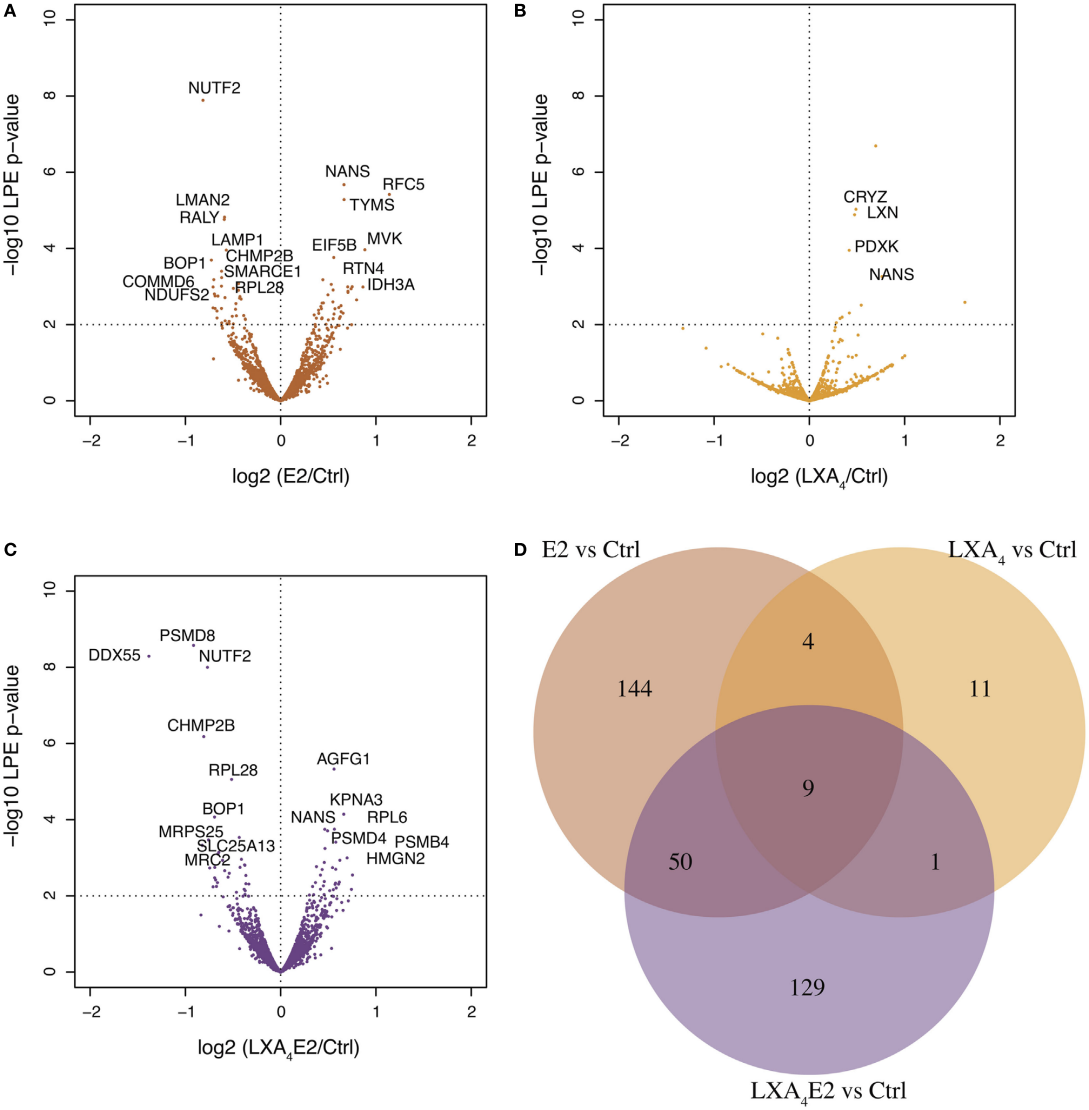
**Volcano plots of proteomic changes induced in 12Z cells treated with (A) 10 nM 17-β-estradiol (E2) versus vehicle, denoted ctrl, (B) 100 nM lipoxin A_4_ (LXA_4_) versus control, and (C) E2/LXA_4_ combination versus control**. The *x*-axis shows the log_2_ ratio of treated sample compared to control while the *y*-axis is (–1)*log_10_ (*p*-value) as calculated using the local pooled error (LPE) test. 348 and 25 significantly differentially expressed proteins were detected, respectively, using the LPE-test (*p*-value <0.05) and a subset with FDR-adjusted *p*-value <0.10 (Benjamini–Hochsberg correction), in one or more comparison (treatment versus control). **(D)** Venn diagram of the 348 significantly differentially expressed proteins in each treated sample versus control. E2 exerted a more marked effect on 12Z endometriotic epithelial cells than LXA_4_. Treatment with a combination of LXA_4_ and E2 resulted in a reduction in the number of proteins impacted.

Using the LPE test, 348 significantly differentially expressed proteins were identified for at least one treatment, with a *p*-value <0.05, while a high quality subset of 27 proteins was identified with a FDR-adjusted *p*-value <0.1, after correction for multiple testing with the BH method. The volcano plot (LPE *p*-value versus log fold change) representation of E2 versus control (Figure 2A), LXA_4_ versus control (Figure 2B), and LXA_4_ in combination with E2 (Figure 2C) provides a general overview of significantly differentially expressed proteins. Many significantly differentially expressed proteins, which passed the LPE test, had a low log_2_ ratio of approximately 0.3. Interestingly, our data suggest that the combination of both treatments exert a suppressive effect on proteins impacted by E2 alone (Figure 2D). The proteins modulated by both LXA_4_ and E2 in combination showed only an overlap of 59 proteins, and 148 proteins that were impacted by E2 alone were no longer significantly impacted in cells treated with the combination. The quantitative information for each significantly differentially expressed protein is shown in Table S2 in Supplementary Material.

**Figure 3:**
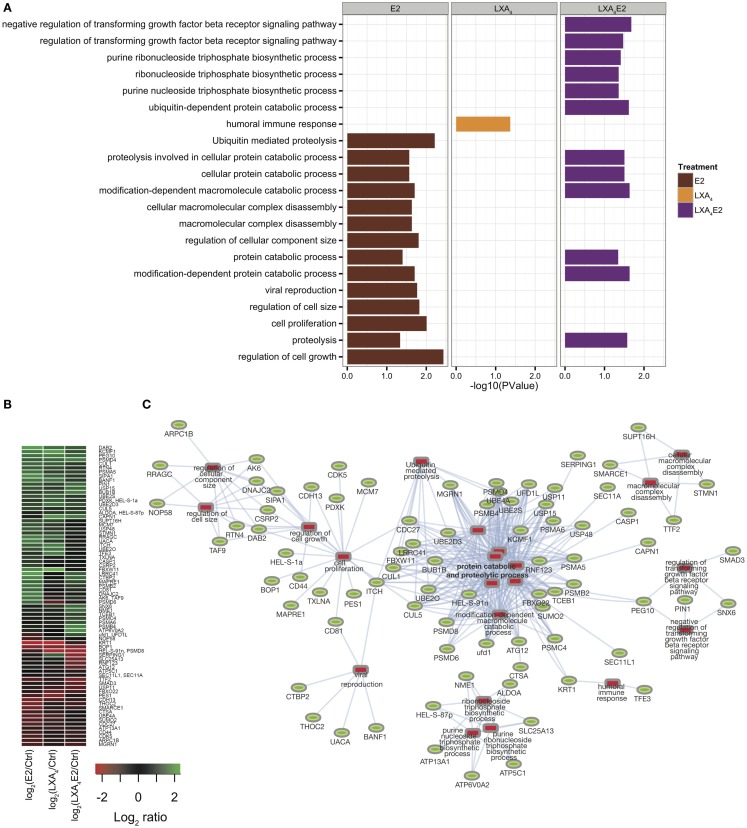
**(A)** Gene ontology analysis using DAVID for each condition versus control. **(B)** Heat map of significantly differentially expressed proteins, having at least one significant GO annotation. **(C)** Protein–annotation network constructed employing the DAVID functional analysis tool and Cytoscape. Significantly enriched GO terms are represented by red rectangles and proteins are represented by green ovals.

The general GO terms for biological processes were retrieved with DAVID (18) for each significantly differentially expressed protein in each treatment condition versus control (Figure 3A, Table S3 in Supplementary Material). Proteins with GO terms linked to cell proliferation, cell death, growth, cell adhesion, and immune processes, as the most relevant to endometriosis pathology, were identified. This analysis demonstrates an antagonist effect of LXA_4_ on E2 signaling at the protein level (Figure 3A). Indeed, LXA_4_ in combination with E2 modulates the cellular macromolecular complex disassembly process, suggesting an inhibition in the growth and proliferation of endometriotic epithelial cells. LXA_4_ alone affects proteins annotated with a single biological process, the humoral immune response, which is perhaps unsurprising as only 25 proteins in total were detected (Figure 2D).

A subsequent quantitative analysis of the 348 protein subset annotated by these GO terms (80 proteins) was performed. The *Z*-score of log ratio of each treatment versus control in 12Z cells is depicted in heat maps using hierarchical clustering for differentially expressed proteins (Figure 3B). Using Cytoscape and GO biological process annotations, a protein–annotation network (in Figure 3C) was modeled, linking our quantitative analysis with protein function.

**Figure 4:**
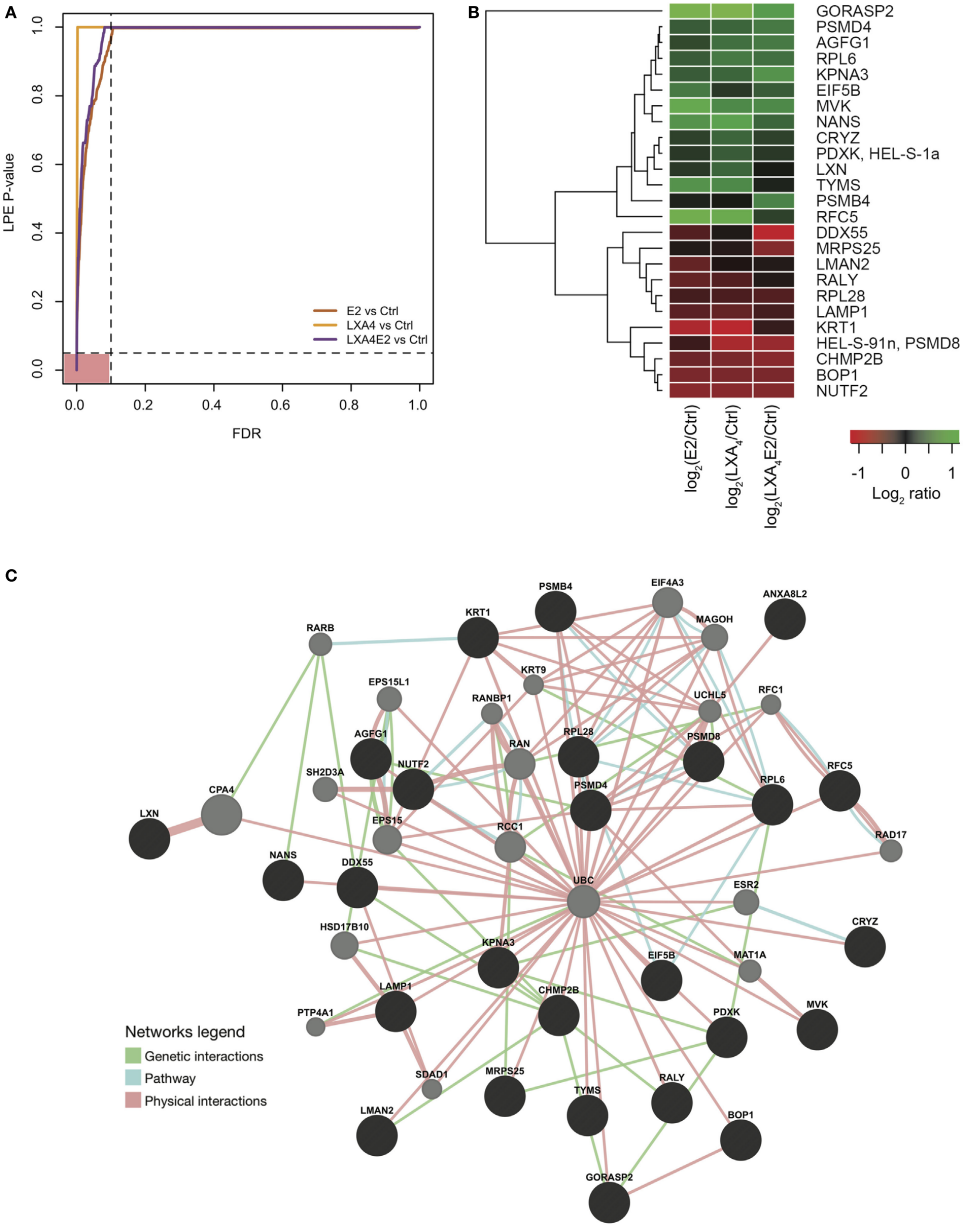
**(A)** FDR control of the LPE-test *p*-values. Significantly regulated proteins obtained from an analysis using a stringently controlled FDR are located in the pink area. **(B)** Heat map of log fold change (treatments versus control) of protein expression with a LPE *p*-value <0.05 and a FDR <0.1. **(C)** Protein–protein interaction network of the significantly differentially expressed proteins (in black), constructed using interactome data from GeneMANIA and Cytoscape. Intermediate nodes (in gray) were added to complete the network, using interactome data and the principle “guilt by association” in GeneMANIA. Genetic interactions (in green), physical interactions (in blue), and pathway interactions (in pink) are presented.

Next, we wished to delineate the contextual relevance using the most recently developed protein–protein interaction bioinformatics resources. The *p*-values of the LPE test as a function of the FDR are presented in Figure 4A. The 27 proteins that fell in the pink area on the graph were selected for network analysis. It can be seen from Figure 4A that a greater number of significantly differentially expressed proteins with a controlled FDR were detected in cells stimulated with LXA_4_E2 versus control and in cells stimulated with E2 versus control. In contrast, a smaller number of significantly differentially expressed proteins with a controlled FDR were detected in cells stimulated with LXA_4_ versus control. Accordingly, the FDR decreases in a steeper manner for cells stimulated with LXA_4_ versus control. The protein–protein interaction network of these 27 significantly differentially expressed proteins with a controlled FDR <0.1, listed in Table 1 (Figures 4A,B) was generated using GeneMANIA^4^ and interactome data from physical interactions, pathway interactions, and genetic interactions (Figure 4C).

**Table 1:** **Significantly differentially expressed proteins according to the LPE test with a FDR <0.1**.

Protein ID	log(E2/ctrl)	log(LXA_4_/ctrl)	log(LXA_4_E2/ctrl)	LPE pval E2 versus ctrl	LPE pval LXA_4_ versus ctrl	LPE pval LXA_4_E2 versus ctrl	FDR E2 versus ctrl	FDR LXA_4_ versus ctrl	FDR LXA_4_E2 versus ctrl	Protein name
P04264	−1.11	−1.33	−0.34	2.35E − 12	1.25E − 02	1.01E − 02	6.85E − 09	1.00E + 00	4.73E − 01	KRT1
Q9H8Y8	1.51	3.30	0.70	4.24E − 12	7.62E − 15	1.00E − 03	6.85E − 09	2.44E − 11	1.71E − 01	GORASP2
P61970	−0.82	−0.79	−0.77	1.29E − 08	1.37E − 01	1.00E − 08	1.39E − 05	1.00E + 00	1.08E − 05	NUTF2
Q9NR45	0.66	0.76	0.46	2.12E − 06	5.50E − 04	1.79E − 04	1.72E − 03	2.96E − 01	5.79E − 02	NANS
P40937	1.14	0.98	0.29	3.83E − 06	7.39E − 02	2.40E − 01	2.48E − 03	1.00E + 00	9.99E − 01	RFC5
P04818	0.67	0.63	0.15	5.25E − 06	1.28E − 01	2.52E − 01	2.83E − 03	1.00E + 00	9.99E − 01	TYMS
Q12907	−0.59	−0.15	−0.23	1.51E − 05	4.70E − 01	4.71E − 02	6.99E − 03	1.00E + 00	8.14E − 01	LMAN2
Q9UKM9	−0.59	−0.50	−0.23	1.74E − 05	3.26E − 01	6.10E − 02	7.04E − 03	1.00E + 00	8.98E − 01	RALY
Q03426	0.88	0.62	0.62	1.09E − 04	2.52E − 01	4.35E − 03	3.58E − 02	1.00E + 00	3.28E − 01	MVK
P11279	−0.57	−0.59	−0.43	1.11E − 04	2.68E − 01	1.78E − 03	3.58E − 02	1.00E + 00	1.99E − 01	LAMP1
O60841	0.56	0.28	0.42	1.73E − 04	5.97E − 01	2.84E − 03	5.09E − 02	1.00E + 00	2.54E − 01	EIF5B
Q14137	−0.73	−0.73	−0.69	2.03E − 04	1.73E − 01	8.54E − 05	5.46E − 02	1.00E + 00	3.45E − 02	BOP1
Q9UQN3	−0.62	−0.70	−0.81	3.97E − 04	1.87E − 01	6.62E − 07	9.88E − 02	1.00E + 00	5.35E − 04	CHMP2B
Q08257	0.29	0.49	0.30	2.81E − 02	9.40E − 06	9.50E − 03	6.48E − 01	1.01E − 02	4.72E − 01	CRYZ
Q9BS40	0.27	0.47	0.06	3.28E − 02	1.31E − 05	5.81E − 01	6.92E − 01	1.06E − 02	9.99E − 01	LXN
O00764	0.28	0.42	0.25	2.40E − 02	1.12E − 04	3.65E − 02	5.96E − 01	7.25E − 02	7.55E − 01	PDXK, HEL-S-1a
P48556	−0.38	−1.08	−0.91	1.23E − 02	4.13E − 02	2.66E − 09	4.25E − 01	1.00E + 00	8.28E − 06	HEL-S-91n, PSMD8
Q8NHQ9	−0.51	−0.16	−1.38	3.47E − 02	7.62E − 01	5.13E − 09	6.97E − 01	1.00E + 00	8.28E − 06	DDX55
P52594	0.36	0.49	0.56	9.85E − 03	6.37E − 02	4.71E − 06	3.93E − 01	1.00E + 00	3.05E − 03	AGFG1
P46779	−0.44	−0.49	−0.51	8.39E − 04	1.75E − 02	8.78E − 06	1.45E − 01	1.00E + 00	4.73E − 03	RPL28
O00505	0.42	0.46	0.66	2.27E − 02	4.00E − 01	7.20E − 05	5.96E − 01	1.00E + 00	3.32E − 02	KPNA3
P55036	0.43	0.47	0.56	6.59E − 03	3.74E − 01	1.78E − 04	3.22E − 01	1.00E + 00	5.79E − 02	PSMD4
Q02878	0.43	0.54	0.49	2.71E − 03	2.23E − 01	1.97E − 04	2.14E − 01	1.00E + 00	5.79E − 02	RPL6
P82663	−0.23	−0.25	−0.76	2.99E − 01	6.40E − 01	3.41E − 04	9.97E − 01	1.00E + 00	8.48E − 02	MRPS25
P28070	0.14	0.06	0.58	4.67E − 01	9.06E − 01	3.90E − 04	9.97E − 01	1.00E + 00	9.01E − 02	PSMB4

*In columns one to three negative values are colored grading from yellow to red and positive values are in green*.

*In columns four to six, the pink color indicates a significant *p*-value for the LPE test and, in columns seven to nine, the yellow color indicates a FDR <0.1*.

The functions of these proteins, as retrieved from Uniprot, are detailed in Table 2. Again, the log_2_ ratio of each treatment divided by control for differentially expressed proteins is depicted in heat maps using hierarchical clustering (Figure 4B).

**Table 2:** **Known functions of significantly differentially expressed proteins with a FDR <0.1 as retrieved from Uniprot**.

Gene names	Function
KRT1 KRTA	May regulate the activity of kinases such as PKC and SRC via binding to integrin beta-1 (ITB1) and the receptor of activated protein kinase C (RACK1/GNB2L1). In complex with C1QBP is a high-affinity receptor for kininogen-1/HMWK
GORASP2 GOLPH6	Plays a role in the assembly and membrane stacking of the Golgi cisternae
NUTF2 NTF2	Facilitates protein transport into the nucleus. Interacts with the nucleoporin p62 and with Ran. Acts at a relatively late stage of nuclear protein import
NANS SAS	Produces *N*-acetylneuraminic acid (Neu5Ac) and 2-keto-3-deoxy-d-glycero-d-galacto-non-onic acid (KDN). Can also use *N*-acetylmannosamine 6-phosphate and mannose 6-phosphate as substrates to generate phosphorylated forms of Neu5Ac and KDN
RFC5	The elongation of primed DNA templates by DNA polymerase delta and epsilon requires the action of the accessory proteins proliferating cell nuclear antigen (PCNA) and activator 1
TYMS TS OK/SW-cl.29	Contributes to the *de novo* mitochondrial thymidylate biosynthesis pathway
LMAN2 C5orf8	Plays a role as an intracellular lectin in the early secretory pathway. Interacts with *N*-acetyl-d-galactosamine and high mannose type glycans and may also bind to O-linked glycans. Involved in the transport and sorting of glycoproteins carrying high mannose type glycans
RALY HNRPCL2 P542	Probable-RNA binding protein. Could be a heterogeneous nuclear ribonucleoprotein (hnRNP). May be involved in pre-mRNA splicing
MVK	May be a regulatory site in the cholesterol biosynthetic pathway
LAMP1	Presents carbohydrate ligands to selectins. Also implicated in tumor cell metastasis
EIF5B IF2 KIAA0741	Function in general translation initiation by promoting the binding of the formylmethionine-tRNA to ribosomes. Seems to function along with eIF-2 (By similarity).
BOP1 KIAA0124	Component of the PeBoW complex, which is required for maturation of 28S and 5.8S ribosomal RNAs and formation of the 60S ribosome
CHMP2B CGI-84	Probable core component of the endosomal sorting required for transport complex III (ESCRT-III) which is involved in multivesicular bodies (MVBs) formation and sorting of endosomal cargo proteins into MVBs. MVBs contain intraluminal vesicles (ILVs) that are generated by invagination and scission from the limiting membrane of the endosome and mostly are delivered to lysosomes enabling degradation of membrane proteins
ANXA8L1 ANXA8L2	Annexin: calcium-dependent phospholipid binding
CRYZ	Does not have alcohol dehydrogenase activity. Binds NADP and acts through a one-electron transfer process. Orthoquinones.
LXN	Hardly reversible non-competitive, and potent inhibitor of CPA1, CPA2, and CPA4. May play a role in inflammation
PDXK	Required for synthesis of pyridoxal-5-phosphate from vitamin B6
PSMD8	Acts as a regulatory subunit of the 26S proteasome which is involved in the ATP-dependent degradation of ubiquitinated proteins. Necessary for activation of the CDC28 kinase
DDX55 KIAA1595	Probable ATP-binding RNA helicase
AGFG1 HRB RAB RIP	Required for vesicle docking or fusion during acrosome biogenesis (By similarity). May play a role in RNA trafficking or localization in case of HIV infection
RPL28	60S ribosomal protein L28
KPNA3 QIP2	Functions in nuclear protein import as an adapter protein for nuclear receptor KPNB1. Binds specifically and directly to substrates containing either a simple or bipartite NLS motif. Docking of the importin/substrate complex to the nuclear pore complex (NPC) is mediated by KPNB1 through binding to nucleoporin FxFG repeats, and the complex is subsequently translocated through the pore by an energy requiring, Ran-dependent mechanism. At the nucleoplasmic side of the NPC, Ran binds to importin-beta and the three components separate and importin-alpha and -beta are re-exported from the nucleus to the cytoplasm where GTP hydrolysis releases Ran from importin. The directionality of nuclear import is thought to be conferred by an asymmetric distribution of the GTP- and GDP-bound forms of Ran between the cytoplasm and nucleus. *In vitro*, mediates the nuclear import of human cytomegalovirus UL84 by recognizing a non-classical NLS. Recognizes NLSs of influenza A virus nucleoprotein probably through ARM repeats 7–9
PSMD4 MCB1	Binds and presumably selects ubiquitin-conjugates for destruction. Displays selectivity for longer polyubiquitin chains. Modulates intestinal fluid secretion
RPL6 TXREB1	Specifically binds to domain C of the Tax-responsive enhancer element in the long terminal repeat of HTLV-I
MRPS25 RPMS25	28S ribosomal protein S25, mitochondrial
PSMB4 PROS26	The proteasome is a multicatalytic proteinase complex which is characterized by its ability to cleave peptides with Arg, Phe, Tyr, Leu, and Glu adjacent to the leaving group at neutral or slightly basic pH. The proteasome has an ATP-dependent proteolytic activity. Mediates the lipopolysaccharide-induced signal macrophage proteasome (by similarity). SMAD1/OAZ1/PSMB4 complex mediates the degradation of the CREBBP/EP300 repressor SNIP1

These 27 proteins interact together to a high degree suggesting their implication in closely related biological processes. Using GeneMANIA, based on the guilt-by-association principle, we completed our query network with 20 interaction partner proteins, which were not detected by the mass spectrometer due to limitations in dynamic range detection but intricately associated with our high quality protein set. ERβ (ESR2) and 17-beta Hydroxysteroid dehydrogenase 10 (17HSDb10), implicated in estrogen metabolism, are among these interaction partner proteins depicted by gray circles in Figure 4C. Basal ERα and ERβ expression by 12Z cells was confirmed by Western blotting prior to performing experiments (data not shown), indicating that these are estrogen-responsive cells.

In this high quality protein subset, the antagonist effect of LXA_4_ on E2 signaling was also observed. LXA_4_ in combination with E2 decreased the expression of GORASP2, NANS, CRYZ, PDXK, LXN, TYMS, RFC5, DDX55, MRPS25, and CHMP2B compared to LXA_4_ or E2 alone. One of the most impacted proteins was Golgi reassembly stacking protein 2 (GORASP2), implicated in Golgi fragmentation and the subsequent entry into mitosis (27) and therefore cell-cycle progression. Similarly, Sialic acid synthase (NANS), which functions in sialic acid biosynthetic pathways (28) and Replication factor C subunit 5 (RFC5), implicated in proliferation, were less up-regulated by LXA_4_E2 than by either treatment alone, suggesting that treating 12Z cells with E2 and LXA_4_ in combination resulted in a decrease of cellular metabolic processes and proliferation.

Lipoxin A_4_ and E2 in combination increased PSMD4, AGFG1, KPNA3, PSMB4, RALY, LAMP1, KRT1, BOP1, and NUTF2 compared to LXA_4_ or E2 alone. LXA_4_ increased the expression of Keratin, type II cytoskeletal 1 (KRT1), which could however also be a contaminant in the lysates. The 26S proteasome non-ATPase regulatory subunit 4 (PSMD4) is involved in antigen processing and presentation of exogenous peptide antigen via MHC class I.

**Figure 5:**
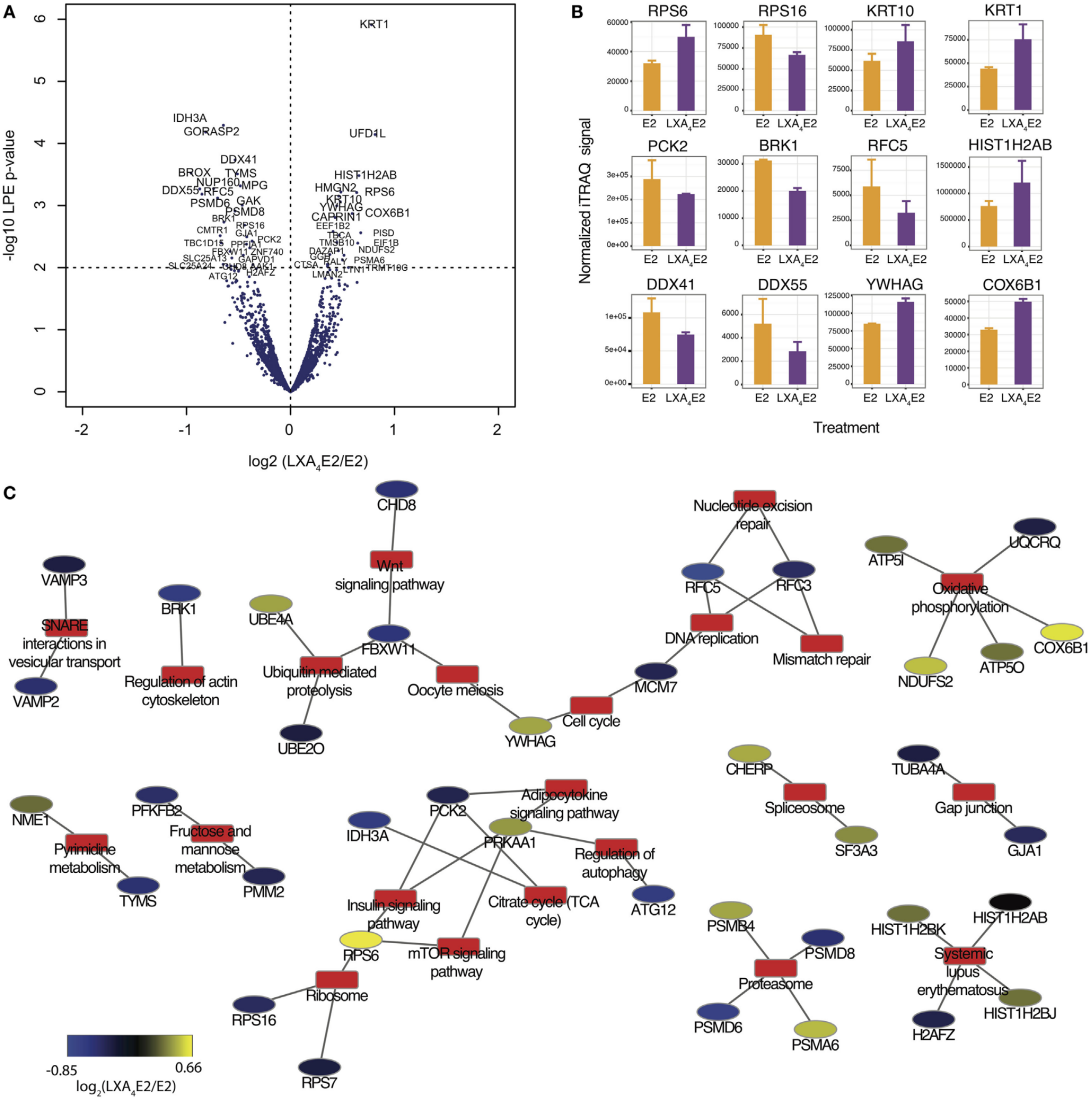
**(A)** Volcano plots of proteomic changes induced in 12Z cells treated with 10 nM E2 and 100 nM LXA_4_ in combination compared with 10 nM E2 alone. **(B)** Selection of significantly differentially expressed proteins with a LPE *p*-value <0.05 and a FDR <0.4. **(C)** Protein–annotation network generated using DAVID, employing KEGG Pathway annotations. Annotations are depicted by red rectangles and proteins are represented by elipses along a blue–yellow color scale of the log_2_ (LXA_4_E2/E2) iTRAQ signal.

With the objective of confirming our observation that LXA_4_ antagonized E2-mediated effects, we compared 12Z cells treated by E2 and LXA_4_ in combination (LXA_4_E2) versus E2 alone. Employing the LPE test, the corresponding volcano plot (Figure 5A) was generated. 146 proteins were significantly differentially expressed in cells treated with the LXA_4_E2 combination versus cells treated with E2 alone (Table S4 in Supplementary Material). Among these proteins, 64 were significantly up-regulated whereas 82 proteins were significantly down-regulated, confirming our hypothesis. There was a 63% overlap between significantly differentially expressed proteins in any of the treatments versus control and LXA_4_E2 versus E2.

Among these 146 proteins (Figure 5B), we found several protein families involved in E2-induced signaling that were impacted by LXA_4_. The DDX, RPS, and KRT families were among those most affected. DEAD box proteins (DDX) are multifunctional proteins, and putative RNA helicases are thought to be involved in cellular growth and division (29). Ribosomal protein S6 (RPS6), a component of the 40S ribosomal subunit, is implicated in mRNA translation. Krt1 and Krt10 are heterodimer partners and members of the type I (acidic) cytokeratin family. Phosphoenolpyruvate carboxykinase 2 (PCK2) expression was attenuated by the LXA_4_E2 combination compared to E2 alone, suggesting a decrease in insulin/glucose metabolism and the TCA cycle, as this enzyme mediates the rate-limiting step in the metabolic pathway that produces glucose from lactate and other precursors derived from the citric acid cycle. The alpha subunit of NAD(+)-specific Isocitrate dehydrogenase 3 (IDH3A) was also markedly affected by LXA_4_E2 compared to E2. These enzymes catalyze the oxidative decarboxylation of isocitrate to alpha-ketoglutarate, the rate-limiting step of the TCA cycle (30).

In order to study the role of these differentially expressed proteins, we subsequently retrieved all pathway annotations (with a minimum of two proteins annotated) using DAVID (Figure 5C). In summary, several pathways of relevance to endometriosis, such as metabolic pathways, notably pyrimidine, fructose, and mannose metabolism, as well as the cell cycle, oxidative phosphorylation, the proteasome, regulation of autophagy, mTOR signaling, and adipocytokines were affected, demonstrating that LXA_4_ modulates several cellular processes which are impacted by E2.

## Discussion

Here, we report the first comprehensive proteomic analysis of changes induced by LXA_4_ and E2 in an endometriotic epithelial cell line. We have performed proteome-wide biomarker and therapeutic target discovery using the most recent bioinformatics tools and databases, coupled with standard statistical analysis.

In order to verify our method, we performed Western blots for CSN5/JAB1, a component of the COP9 signalosome, a complex which regulates several cellular and developmental processes (31), and observed that this protein were significantly increased in LXA_4_E2 conditions versus control. ERα was previously shown to co-immuno-precipitate with CSN5 and overexpression of CSN5 caused an increase in ligand-induced ERα degradation (32), indicating the functional relevance of this observation. There was a high degree of correlation between the Western blot and the MS data, confirming the robustness of our results.

Most proteins whose expression was induced by LXA_4_, were also induced by E2, as would be expected from our previous observations in endometrial epithelial cells where we characterized this eicosanoid as an estrogen receptor agonist which bound ER, activated canonical ER signaling and also inhibited E2-mediated responses including gene expression and cellular proliferation (8). As observed in that previous study where LXA_4_ exhibited less potent responses that E2, the natural ligand, in the present study, fewer proteins are regulated by LXA_4_ than by E2, and LXA_4_ also inhibited certain E2-mediated changes in protein expression.

Elevated local E2 levels and increased expression of estrogen-regulated molecules, which promote proliferation, are features of endometriotic lesions (33–36), and molecules that inhibit estrogen production and/signaling represent potential therapeutics. We and others have demonstrated that LXA_4_ and its analogs have decreased endometriosis progression in rodent models and may represent future therapies (11, 37, 38). The latter study reported attenuated expression of estrogen-regulated mediators implicated in cell proliferation in mice with endometriosis treated with LXA_4_ compared to vehicle-treated controls.

It is perhaps unsurprising that LXA_4_, as a molecule that acts via several known receptors notably ALX/FPR2 and ERα, also impacts diverse pathways as well as metabolism. For example, Mevalonate kinase (MVK), a key early enzyme in isoprenoid and sterol synthesis and therefore in cholesterol production via the generation of Squalene (39), exhibited a 25% reduced induction in cells stimulated with E2 and LXA_4_ in combination compared to cells treated with E2 alone. The Mevalonate pathway is a key metabolic pathway as its blockade has been linked to mitochondrial dysfunction and defective autophagy, possibly causing inflammasome activation and subsequent cell death (40). This antagonistic modulation of E2-driven responses by LXA_4_, also observed for several other proteins, is consistent with the notion that the regulation of these proteins is mediated via ER, or by another common receptor.

The LXA_4_E2 combination appeared to attenuate insulin/glucose metabolism and the TCA cycle by impacting IDH3A and PCK2, the latter enzyme was already previously studied in decidualized stromal endometrial cells (41) but its role in endometriotic epithelial cells remains unclear.

RPS6, another protein detected, is a component of the 40S ribosomal subunit and is therefore thought to be involved in regulating translation. While its precise function is currently under investigation, studies have shown that RPS6 is involved in the regulation of cell size, cell proliferation, and glucose homeostasis (42). RPS6 has been implicated in mTOR signaling in hormone responsive cells (43, 44). Of note, E2 has been shown to trigger protein synthesis in mouse uterine epithelial cells via the PKC-ERK1/2–mTOR pathway (45). Furthermore, the PI3K/mTOR/AKT pathway is activated in endometriosis, and inhibition of this pathway represents a potential therapeutic modality (46, 47). Consistent with our previous studies (8), both LXA_4_ and E2 appear to increase cellular proliferation individually but in combination, this effect appears to be blunted, which may be occurring via crosstalk between the ER and mTOR pathways. Indeed, it is recently appreciated that such crosstalk mechanisms exist in cancer cells (48).

Our study also provides novel insights into common intracellular signaling pathways activated by LXA_4_ and E2, for example those involved in protein synthesis. eIF5B, reported to be the most catalytically active of the translation initiation factors (49), was one of the top 25 proteins regulated. Interestingly, this protein was previously demonstrated to be increased by E2 in MCF-7 estrogen-responsive breast cancer cells via ER (50).

Though a cell line cannot reproduce complex tissue conditions, 12Z cells proved useful to optimize the technique, and the data generated will allow the study of potential new therapeutic targets or endometriosis biomarkers. However, proteomic methods are still characterized by some heterogeneity, and further standardization and optimization for clinical use is necessary, especially at the sample preparation level to extract proteins from urine, menstrual blood, or other body fluids (51, 52). Although the effects of the different treatments are modest and our experimental design includes only two replicates, which limits our statistical power, we found several relevant processes implicated in endometriosis physiopathology. The proteins identified in this study require further validation in order to be considered as potential therapeutic targets.

Emerging fractionation techniques to capture and concentrate low-abundance proteins, new non-gel-based proteomic technologies combined with protein labeling strategies and the ongoing development of advanced bioinformatics tools will hopefully allow the development of relatively non-invasive diagnostic methods for endometriosis. This is a major unmet medical need as it would facilitate earlier diagnosis of this common, chronic disease.

## Author Contributions

JS: Study design, sample preparation, data analysis, bioinformatics, and manuscript writing. PW: Study design, mass spectrometry expertise, critical discussion, and manuscript corrections. IG: Western blot, cell culture, and manuscript corrections. MQ: study design, mass spectrometry expertise, critical discussion, and manuscript corrections. GC: study conception and design, data analysis, critical discussion, and manuscript writing.

## Conflict of Interest Statement

The authors declare that the research was conducted in the absence of any commercial or financial relationships that could be construed as a potential conflict of interest.

## Footnotes

^1^www.uniprot.org

^2^https://david.ncifcrf.gov

^3^http://www.cytoscape.org

^4^http://www.genemania.org

## References

[1] Giudice LC Kao LC. Endometriosis. Lancet (2004) 364:1789–99. 10.1016/S0140-6736(04)17403-5 15541453

[2] Agarwal N Subramanian A. Endometriosis – morphology, clinical presentations and molecular pathology. J Lab Physicians (2010) 2:1–9. 10.4103/0974-2727.66699 21814398 PMC3147077

[3] Nnoaham KE Hummelshoj L Webster P d’Hooghe T de Cicco Nardone F de Cicco Nardone C Impact of endometriosis on quality of life and work productivity: a multicenter study across ten countries. Fertil Steril (2011) 96:366–373e8. 10.1016/j.fertnstert.2011.05.090 21718982 PMC3679489

[4] Fuldeore M Yang H Du EX Soliman AM Wu EQ Winkel C. Healthcare utilization and costs in women diagnosed with endometriosis before and after diagnosis: a longitudinal analysis of claims databases. Fertil Steril (2015) 103:163–71. 10.1016/j.fertnstert.2014.10.011 25455535

[5] Bulun SE Endometriosis. N Engl J Med (2009) 360:268–79. 10.1056/NEJMra0804690 19144942

[6] Serhan CN. Pro-resolving lipid mediators are leads for resolution physiology. Nature (2014) 510:92–101. 10.1038/nature13479 24899309 PMC4263681

[7] Serhan CN Chiang N Van Dyke TE. Resolving inflammation: dual anti-inflammatory and pro-resolution lipid mediators. Nat Rev Immunol (2008) 8:349–61. 10.1038/nri2294 18437155 PMC2744593

[8] Russell R Gori I Pellegrini C Kumar R Achtari C Canny GO. Lipoxin A4 is a novel estrogen receptor modulator. FASEB J (2011) 25:4326–37. 10.1096/fj.11-187658 21885654

[9] Clark JH Markaverich BM. The agonistic and antagonistic actions of estriol. J Steroid Biochem (1984) 20:1005–13. 10.1016/0022-4731(84)90011-6 6202959

[10] Melamed M Castano E Notides AC Sasson S. Molecular and kinetic basis for the mixed agonist/antagonist activity of estriol. Mol Endocrinol (1997) 11:1868–78. 10.1210/mend.11.12.0025 9369454

[11] Kumar R Clerc AC Gori I Russell R Pellegrini C Govender L Lipoxin A(4) prevents the progression of de novo and established endometriosis in a mouse model by attenuating prostaglandin E(2) production and estrogen signaling. PLoS One (2014) 9:e89742 10.1371/journal.pone.0089742 24587003 PMC3933674

[12] Zeitvogel A Baumann R Starzinski-Powitz A. Identification of an invasive, N-cadherin-expressing epithelial cell type in endometriosis using a new cell culture model. Am J Pathol (2001) 159:1839–52. 10.1016/S0002-9440(10)63030-1 11696444 PMC1867070

[13] Grund EM Kagan D Tran CA Zeitvogel A Starzinski-Powitz A Nataraja S Tumor necrosis factor-alpha regulates inflammatory and mesenchymal responses via mitogen-activated protein kinase kinase, p38, and nuclear factor kappaB in human endometriotic epithelial cells. Mol Pharmacol (2008) 73:1394–404. 10.1124/mol.107.042176 18252806

[14] Banu SK Lee J Starzinski-Powitz A Arosh JA. Gene expression profiles and functional characterization of human immortalized endometriotic epithelial and stromal cells. Fertil Steril (2008) 90:972–87. 10.1016/j.fertnstert.2007.07.1358 18001719

[15] Lee J Banu SK Subbarao T Starzinski-Powitz A Arosh JA. Selective inhibition of prostaglandin E2 receptors EP2 and EP4 inhibits invasion of human immortalized endometriotic epithelial and stromal cells through suppression of metalloproteinases. Mol Cell Endocrinol (2011) 332:306–13. 10.1016/j.mce.2010.11.022 21111772

[16] Pichler P Kocher T Holzmann J Mazanek M Taus T Ammerer G Peptide labeling with isobaric tags yields higher identification rates using iTRAQ 4-plex compared to TMT 6-plex and iTRAQ 8-plex on LTQ Orbitrap. Anal Chem (2010) 82:6549–58. 10.1021/ac100890k 20593797 PMC3093924

[17] Unwin RD Quantification of proteins by iTRAQ. Methods Mol Biol (2010) 658:205–15. 10.1007/978-1-60761-780-8_12 20839106

[18] Huang da W Sherman BT Lempicki RA. Systematic and integrative analysis of large gene lists using DAVID bioinformatics resources. Nat Protoc (2009) 4:44–57. 10.1038/nprot.2008.211 19131956

[19] Ashburner M Ball CA Blake JA Botstein D Butler H Cherry JM Gene ontology: tool for the unification of biology. The Gene Ontology Consortium. Nat Genet (2000) 25:25–9. 10.1038/75556 10802651 PMC3037419

[20] Killcoyne S Carter GW Smith J Boyle J. Cytoscape: a community-based framework for network modeling. Methods Mol Biol (2009) 563:219–39. 10.1007/978-1-60761-175-2_12 19597788

[21] Shannon P Markiel A Ozier O Baliga NS Wang JT Ramage D Cytoscape: a software environment for integrated models of biomolecular interaction networks. Genome Res (2003) 13:2498–504. 10.1101/gr.1239303 14597658 PMC403769

[22] Geiser L Dayon L Vaezzadeh AR Hochstrasser DF. Shotgun proteomics: a relative quantitative approach using Off-Gel electrophoresis and LC-MS/MS. Methods Mol Biol (2011) 681:459–72. 10.1007/978-1-60761-913-0_27 20978983

[23] Ting L Cowley MJ Hoon SL Guilhaus M Raftery MJ Cavicchioli R. Normalization and statistical analysis of quantitative proteomics data generated by metabolic labeling. Mol Cell Proteomics (2009) 8:2227–42. 10.1074/mcp.M800462-MCP200 19605365 PMC2758752

[24] Jain N Thatte J Braciale T Ley K O’Connell M Lee JK. Local-pooled-error test for identifying differentially expressed genes with a small number of replicated microarrays. Bioinformatics (2003) 19:1945–51. 10.1093/bioinformatics/btg264 14555628

[25] Mostafavi S Ray D Warde-Farley D Grouios C Morris Q. GeneMANIA: a real-time multiple association network integration algorithm for predicting gene function. Genome Biol (2008) 9(Suppl 1):S4. 10.1186/gb-2008-9-s1-s4 18613948 PMC2447538

[26] Kato JY Yoneda-Kato N. Mammalian COP9 signalosome. Genes Cells (2009) 14:1209–25. 10.1111/j.1365-2443.2009.01349.x 19849719

[27] Duran JM Kinseth M Bossard C Rose DW Polishchuk R Wu CC The role of GRASP55 in Golgi fragmentation and entry of cells into mitosis. Mol Biol Cell (2008) 19:2579–87. 10.1091/mbc.E07-10-0998 18385516 PMC2397314

[28] Lawrence SM Huddleston KA Pitts LR Nguyen N Lee YC Vann WF Cloning and expression of the human N-acetylneuraminic acid phosphate synthase gene with 2-keto-3-deoxy-D-glycero-D-galacto-nononic acid biosynthetic ability. J Biol Chem (2000) 275:17869–77. 10.1074/jbc.M000217200 10749855

[29] Linder P Jankowsky E. From unwinding to clamping – the DEAD box RNA helicase family. Nat Rev Mol Cell Biol (2011) 12:505–16. 10.1038/nrm3154 21779027

[30] Sazanov LA Jackson JB. Proton-translocating transhydrogenase and NAD- and NADP-linked isocitrate dehydrogenases operate in a substrate cycle which contributes to fine regulation of the tricarboxylic acid cycle activity in mitochondria. FEBS Lett (1994) 344:109–16. 10.1016/0014-5793(94)00370-X 8187868

[31] Wei N Serino G Deng XW. The COP9 signalosome: more than a protease. Trends Biochem Sci (2008) 33:592–600. 10.1016/j.tibs.2008.09.004 18926707

[32] Callige M Kieffer I Richard-Foy H CSN5/Jab1 is involved in ligand-dependent degradation of estrogen receptor {alpha} by the proteasome. Mol Cell Biol (2005) 25:4349–58. 10.1128/MCB.25.11.4349-4358.2005 15899841 PMC1140630

[33] Noble LS Simpson ER Johns A Bulun SE Aromatase expression in endometriosis. J Clin Endocrinol Metab (1996) 81:174–9. 10.1210/jc.81.1.174 8550748

[34] Bulun SE Monsivais D Kakinuma T Furukawa Y Bernardi L Pavone ME Molecular biology of endometriosis: from aromatase to genomic abnormalities. Semin Reprod Med (2015) 33:220–4. 10.1055/s-0035-1554053 26036904

[35] Canny GO Lessey BA. The role of lipoxin A4 in endometrial biology and endometriosis. Mucosal Immunol (2013) 6:439–50. 10.1038/mi.2013.9 23485944 PMC4062302

[36] Pellegrini C Gori I Achtari C Hornung D Chardonnens E Wunder D The expression of estrogen receptors as well as GREB1, c-MYC, and cyclin D1, estrogen-regulated genes implicated in proliferation, is increased in peritoneal endometriosis. Fertil Steril (2012) 98:1200–8. 10.1016/j.fertnstert.2012.06.056 22884659

[37] Xu Z Zhao F Lin F Chen J Huang Y. Lipoxin A4 inhibits the development of endometriosis in mice: the role of anti-inflammation and anti-angiogenesis. Am J Reprod Immunol (2012) 67:491–7. 10.1111/j.1600-0897.2011.01101.x 22229383

[38] Chen QH Zhou WD Pu DM Huang QS Li T Chen QX. 15-Epi-lipoxin A(4) inhibits the progression of endometriosis in a murine model. Fertil Steril (2010) 93:1440–7. 10.1016/j.fertnstert.2009.01.107 19268934

[39] Edwards PA Ericsson J. Sterols and isoprenoids: signaling molecules derived from the cholesterol biosynthetic pathway. Annu Rev Biochem (1999) 68:157–85. 10.1146/annurev.biochem.68.1.157 10872447

[40] Tricarico PM Crovella S Celsi F. Mevalonate pathway blockade, mitochondrial dysfunction and autophagy: a possible link. Int J Mol Sci (2015) 16:16067–84. 10.3390/ijms160716067 26184189 PMC4519939

[41] Bombail V Gibson DA Collins F MacPherson S Critchley HO Saunders PT. A role for the orphan nuclear receptor estrogen-related receptor alpha in endometrial stromal cell decidualization and expression of genes implicated in energy metabolism. J Clin Endocrinol Metab (2010) 95:E224–8. 10.1210/jc.2010-0154 20668045 PMC3050102

[42] Ruvinsky I Sharon N Lerer T Cohen H Stolovich-Rain M Nir T Ribosomal protein S6 phosphorylation is a determinant of cell size and glucose homeostasis. Genes Dev (2005) 19:2199–211. 10.1101/gad.351605 16166381 PMC1221890

[43] Volovelsky O Cohen G Kenig A Wasserman G Dreazen A Meyuhas O Phosphorylation of ribosomal protein S6 mediates mammalian target of rapamycin complex 1-induced parathyroid cell proliferation in secondary hyperparathyroidism. J Am Soc Nephrol (2015). 10.1681/ASN.2015040339 26283674 PMC4814192

[44] Li G Shan C Liu L Zhou T Zhou J Hu X Tanshinone IIA inhibits HIF-1alpha and VEGF expression in breast cancer cells via mTOR/p70S6K/RPS6/4E-BP1 signaling pathway. PLoS One (2015) 10:e0117440 10.1371/journal.pone.0117440 25659153 PMC4320086

[45] Wang Y Zhu L Kuokkanen S Pollard JW Activation of protein synthesis in mouse uterine epithelial cells by estradiol-17beta is mediated by a PKC-ERK1/2-mTOR signaling pathway. Proc Natl Acad Sci U S A (2015) 112:E1382–91. 10.1073/pnas.1418973112 25733860 PMC4371960

[46] Makker A Goel MM Das V Agarwal A. PI3K-Akt-mTOR and MAPK signaling pathways in polycystic ovarian syndrome, uterine leiomyomas and endometriosis: an update. Gynecol Endocrinol (2012) 28:175–81. 10.3109/09513590.2011.583955 21916800

[47] Leconte M Nicco C Ngo C Chereau C Chouzenoux S Marut W The mTOR/AKT inhibitor temsirolimus prevents deep infiltrating endometriosis in mice. Am J Pathol (2011) 179:880–9. 10.1016/j.ajpath.2011.04.020 21718677 PMC3157265

[48] Andruska ND Zheng X Yang X Mao C Cherian MM Mahapatra L Estrogen receptor alpha inhibitor activates the unfolded protein response, blocks protein synthesis, and induces tumor regression. Proc Natl Acad Sci U S A (2015) 112:4737–42. 10.1073/pnas.1403685112 25825714 PMC4403155

[49] Merrick WC. Mechanism and regulation of eukaryotic protein synthesis. Microbiol Rev (1992) 56:291–315. 1620067 10.1128/mr.56.2.291-315.1992 PMC372869

[50] Garcia SA Nagai MA Transcriptional regulation of bidirectional gene pairs by 17-beta-estradiol in MCF-7 breast cancer cells. Braz J Med Biol Res (2011) 44:112–22. 10.1590/S0100-879X2010007500149 21180879

[51] Yang H Zhou B Prinz M Siegel D Proteomic analysis of menstrual blood. Mol Cell Proteomics (2012) 11:1024–35. 10.1074/mcp.M112.018390 22822186 PMC3494145

[52] Marimuthu A O’Meally RN Chaerkady R Subbannayya Y Nanjappa V Kumar P A comprehensive map of the human urinary proteome. J Proteome Res (2011) 10:2734–43. 10.1021/pr2003038 21500864 PMC4213861

