# Supplementary figures and images for: Proteome-Wide Effect of 17-β-Estradiol and Lipoxin A4 in an Endometriotic Epithelial Cell Line
Source: Front Endocrinol (Lausanne). 2016 Jan 6;6:192. doi: 10.3389/fendo.2015.00192 (PMC4701930; doi:10.3389/fendo.2015.00192)

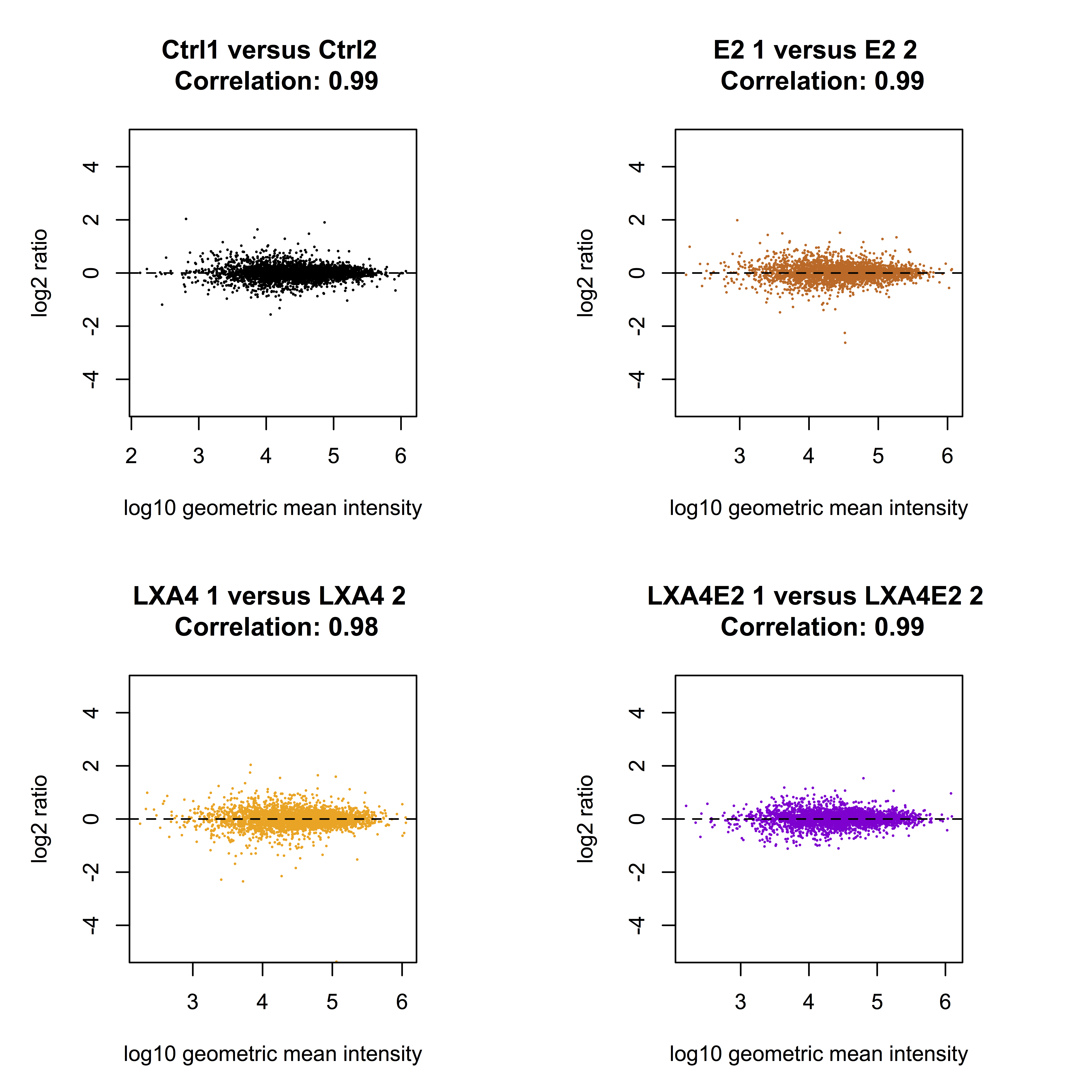

Supplement: Supplementary file 5 [file Image_1.JPEG]
